# Impacts of feral horses on a desert environment

**DOI:** 10.1186/1472-6785-9-22

**Published:** 2009-11-10

**Authors:** Stacey D Ostermann-Kelm, Edward A Atwill, Esther S Rubin, Larry E Hendrickson, Walter M Boyce

**Affiliations:** 1 Wildlife Health Center, University of California, Davis, CA 95616, USA; 2 School of Veterinary Medicine, University of California, Davis, CA 95616, USA; 3 Conservation Biology Institute, PO Box 369, Borrego Springs, CA 92004, USA; 4 Anza-Borrego Desert State Park, Borrego Springs, CA 92004, USA

## Abstract

**Background:**

Free-ranging horses (*Equus caballus*) in North America are considered to be feral animals since they are descendents of non-native domestic horses introduced to the continent. We conducted a study in a southern California desert to understand how feral horse movements and horse feces impacted this arid ecosystem. We evaluated five parameters susceptible to horse trampling: soil strength, vegetation cover, percent of nonnative vegetation, plant species diversity, and macroinvertebrate abundance. We also tested whether or not plant cover and species diversity were affected by the presence of horse feces.

**Results:**

Horse trailing resulted in reduced vegetation cover, compacted soils, and in cases of intermediate intensity disturbance, increased plant species diversity. The presence of horse feces did not affect plant cover, but it did increase native plant diversity.

**Conclusion:**

Adverse impacts, such as soil compaction and increased erosion potential, were limited to established horse trails. In contrast, increased native plant diversity near trails and feces could be viewed as positive outcomes. Extensive trailing can result in a surprisingly large impact area: we estimate that < 30 horses used > 25 km^2 ^of trails in our study area.

## Background

Zoogeomorphology, the study of the geomorphologic impacts of animals [1], is a relatively new field of research. Ecologists and geomorphologists are placing increasing emphasis on understanding the fundamental role of animals as agents of erosion, transportation, and deposition of sediment [2,3]. Zoogeomorphologic data may be useful to wildlife managers who need to quantify, predict, and manage impacts from feral species on native ecosystems.

Zoogeomorphology may be particularly helpful in understanding the controversial role of feral horses (*Equus caballus*) in native ecosystems [4-8]. Feral horses have a wide geographic range across the southwestern United States and may potentially affect many species through seemingly small changes to the ecosystems they inhabit. For instance, the tendency for feral horses to use landscapes heterogeneously results in the creation of multiple trails [5]. We documented an extensive complex of trails that were used and maintained by feral horses in southern California and similar trail complexes have been documented in Nevada [5]. Horse feces and trails may visually impair the landscape and detract from the experience of some wilderness users. Natural animal trails from a variety of species are well documented, primarily through casual mention in the literature, but little quantitative information on the impacts of animal trails in natural environments exists [3,9]

Trampling, which leads to the formation of trails, is one of the most influential geomorphologic effects of large mammals such as feral horses [3]. Through a variety of mechanisms, trampling may lead directly or indirectly to erosion [7]. It is a direct agent of erosion when hoof chiseling leads directly to erosion. Trampling is an indirect agent of erosion when it prepares the soil for erosion through other geomorphic processes; for example, when trampling removes vegetative cover or increases soil bulk density (i.e., causes soil compaction). Soil compaction is problematic because it increases soil strength, reduces both rainwater infiltration rates and soil pore volume [3], and affects plant root growth through increased mechanical impedance and elevated soil temperatures [10]. Reducing vegetative cover increases the likelihood of soil erosion.

Erosion is of special concern for desert soils because nutrients are often concentrated in the surface soil [11]. Even the loss of a few centimeters of surface soil may substantially reduce available nutrients for plants and disrupt community structure [12]. Microbiotic soil crusts serve important functions in soil stabilization, nitrogen fixation, and water conservation. These crusts are concentrated in the top 1-4 mm of soil and are particularly sensitive to trampling [11].

Erosion, soil compaction, and vegetation loss from trampling can ultimately alter the structure of local soil, plant, macroinvertebrate, bird, or small mammal communities [3,6,7,11,13]. Soil disturbance and compaction have been associated with decreased plant germination [11]. Decreases in plant cover have been associated with decreases in the diversity and abundance of lizards [11] and increased predation rates on bird eggs [14]. Because geomorphic changes may have cascading effects that potentially extend to multiple species, it is important to quantify the geomorphic impacts of large, wide-ranging mammals such as feral horses. The impacts of cattle trampling and grazing on native plant and animal species have been relatively well-studied. However, because of behavioral, morphological and evolutionary differences, it is not appropriate to assume that impacts from feral horses are similar to those of domestic livestock [5,15].

In this study our first objective was to quantify, at varying distances from trails used by horses, five parameters susceptible to horse trampling: soil strength, vegetation cover, percent of nonnative vegetation, plant species diversity, and macroinvertebrate abundance. Another potentially important effect of feral horses in an arid environment is their role, via feces, in redistributing seeds, nutrients, and moisture, as well as increasing soil pH [16]. An adult horse deposits an estimated 9145 kg of feces per year [17], and the extra nutrients and moisture provided by horse feces may provide a more favorable environment for both native and nonnative plant species. Our second objective in this study was to quantify changes in plant cover and species diversity near feral horse feces.

## Methods

### Study Area

We sampled upland habitat near Upper Willows and Alder Canyon at the northwest end of Coyote Canyon in Anza-Borrego Desert State Park (ABDSP), California, USA. Coordinates for the approximate center of our study area are latitude 33.438, longitude -116.523. Coyote Canyon is within the Colorado subdivision of the Sonoran Desert. This area was chosen for sampling because it was a high use area for feral horses and it contained a dense complex of visible trails used by feral horses.

Soils in the study area are of granitic origin and range in texture and composition from course sand to fine sandy loam, and in some cases, fine silt. Vegetation in Coyote Canyon is dominated by chaparral at elevations above approximately 1500 m and by pinyon pine (*Pinus monophylla*)-juniper (*Juniperus californica*) above approximately 1200 m. At lower elevations, vegetation is dominated by agave (*Agave deserti*), ocotillo (*Fouquieria splendens*), cholla (*Opuntia *spp.), palo verde (*Cercidium floridum*), creosote (*Larrea tridentata*), and palo verde-mesquite (*Prosopis *spp.) associations. Annual species comprise over half the flora in the Colorado subdivision of the Sonoran Desert and are mostly winter growing species that flourish only in wet years.

Climate is characterized by hot summers, cool wet winters, and a bimodal precipitation pattern, with most precipitation occurring during November - February and a smaller amount occurring during July - September. Between 1948 and 2003, annual precipitation averaged 149.1 mm, but was only 29.7 mm and 118.6 mm in 2002 and 2003, respectively (Western Regional Climate Center, Borrego Desert Park Station). This area of the Sonoran Desert is classified as arid [18].

Historically, Coyote Canyon was home to several bands of Native Americans, followed by a small number of cattlemen and homesteaders between the 1880s and 1960s. Feral cattle were introduced into the canyon during the Anza Expeditions beginning in 1773. Cattle ranchers and the Los Coyotes Indians maintained horses and cattle in Coyote Canyon beginning in the mid-1800s and into the early 1900s. Small numbers of feral cattle roamed the canyon until they were captured and removed in 1987.

A population of approximately 20-40 feral horses inhabited Coyote Canyon from circa-1920 until March 2003. Anecdotal accounts suggested the horses were released from a nearby ranch in the 1920s, but the actual origin and date of introduction of these horses is unknown. In March 2003, California State Parks removed all feral horses (*n *= 29) from the canyon due to poor range conditions. Young and female horses were translocated to a wild horse sanctuary and the males were transferred to a Bureau of Land Management holding facility.

It is important to note that our study area was subject to cattle grazing for approximately 100 years. One of the more difficult aspects of researching feral horse impacts on arid environments is finding a study site that was not previously grazed by cattle. The rarity of ungrazed land in the southwestern U.S. effectively forces researchers to use previously grazed lands in studies of other land impacts [6,7,11]. While cattle were removed from Coyote Canyon >15 years before our study, 15 years is probably insufficient time to allow complete recovery of vegetation to pre-grazing conditions [11]. Therefore, although we defined horse trails as those trails apparently used and maintained by feral horses in the 15 years prior to our study, it is possible that many of these trails were originally created or used by cattle. The feral horse trails sampled in this study are distinct and separate from the equestrian riding trail in Coyote Canyon that is maintained by California State Parks. Wild burros (*Equus asinus*) are found in a few locations in southern California but were absent from our site.

### Field sampling

Using ARCVIEW 3.2 (Environmental Systems Research Institute, Inc., Redlands, California), we created a study area polygon within Coyote Canyon that encompassed approximately 10 km^2 ^of upland habitat used frequently by feral horses. We chose this area to provide a relatively homogeneous study area that did not include riparian vegetation. Using ARCVIEW, we generated 120 randomly placed potential starting points within the polygon. From each of our starting points, we walked a spiral circle outward to the first encountered horse trail. We considered only obvious paths ≥ 30 cm wide containing <25% vegetation cover for a linear distance of >10 m, and/or linear paths showing clear incision as starting points for sampling. Once a clear trail was identified, whenever possible, we added three additional sampling points along the trail, spaced 120 paces (approximately 50 m) apart. In these cases, trails may not have measured >30 cm in width where sampling occurred.

At each trail sampling point, we recorded the trail width and depth (measured as the difference between the center of the trail and a meter stick placed perpendicular to the trail, resting on the edges of the trail). Two 6.4 m transects were established perpendicular to each side of the trail. We placed 30 cm^2 ^quadrat frames in the center of the trail and at 0, 0.40, 1.40, and 6.40 m from each side of the trail, along the transect, resulting in nine quadrats per trail sampling site. Quadrats at 0 m from the trail were immediately adjacent to the edge of the trail. Quadrats at 6.40 m from the edge of the trail were designated as the referent or control plots because we expected trail impacts to be minimal >6 m from the trail. If a referent plot fell on or near (within a 5-m radius) a horse trail, the quadrat was relocated to a nearby area (within 10 m) not having feral horse trailing. The exact placement of the quadrat at the relocated site was determined by tossing the quadrat frame in the designated area. Horse fecal piles were selected for sampling by searching a 15-m diameter area near referent quadrats. Only feces estimated to be <1-year-old based on moisture content and color were sampled. A 30-cm^2 ^quadrat was placed immediately adjacent to the feces in one of the four, systematically-altered cardinal directions.

For all 1080 quadrats, we estimated the total percent cover and the percent cover of each plant species. Cover was estimated as the actual percentage of the quadrat covered by vegetation rooted within the quadrat, without rounding or filling in areas between leaves or vegetative parts. Plant species were identified and classified as native or non-native [19]. For each quadrat, we also recorded whether the referent quadrat was moved (described above), the percentage of the quadrat under the dripline of a shrub, and the presence or absence of horse feces.

We counted all live macroinvertebrates (e.g., Araneae, Coleoptera, Hemiptera, Hymenoptera) within a quadrat during a 10-s scan within quadrats at the trail center, and at 0, 0.40 and 6.40 m from the trail. Vegetation was manipulated when necessary to improve visibility. We also measured soil strength eight times per quadrat in quadrats at the trail center, and at 0, 0.40, and 6.40 m. Soil strength is a measure used to characterize soil compaction. We measured soil strength in kg/cm^2 ^using a hand-held pocket penetrometer (ELE International Pocket Penetrometer Model 29-3729, Pelham, AL, USA). For consistency, all soil measurements were made by one person using the same penetrometer. Macroinvertebrate and soil data were not collected from quadrats at 1.40 m from the trail or for those adjacent to horse feces, in order to improve data collection efficiency and facilitate a two-person field team. Soil strength data was not collected during the first week of fieldwork because the equipment was not available.

We estimated the total length of trails used by feral horses in the greater Coyote Canyon area by viewing aerial photographs of Coyote Canyon http://terraserver-usa.com/ and measuring visible trails using TOPO 2.3.2 (Digital Data Services, Inc., Lakewood, CO, USA). We also recorded some of the less-visible trails via GPS during field surveys and later displayed and measured these trails using TOPO. As a result of our methodology and incomplete survey of the canyon, our estimate of the trails used by feral horses in Coyote Canyon represents a minimum estimate.

### Data Analysis

Data were analyzed using S-PLUS 2000 Professional Release 2 (MathSoft, Inc). We used linear mixed-effects models [20] for continuous outcome variables and Poisson regression models [21] for discrete outcome variables. Outcome variables included percent total plant cover, percent cover of nonnative plant species, number of native plant species, soil strength, and number of macroinvertebrates. Continuous covariates were distance from the trail (QUADRAT), percent of the quadrat under a shrub dripline (DRIPLINE), day of data collection (DAY), trail area (width × depth in mm; TRAILAREA), and average soil strength (SOIL). Categorical covariates were presence or absence of precipitation within the preceding 48 hrs (RAIN), presence or absence of horse feces or tracks within the quadrat (HORSESIGN), and whether or not the referent quadrat was moved to avoid a trail (MOVED). The referent conditions, to which all models were compared, included no rain in the preceding 48 hours, absence of horse feces and tracks, and "not moved" for the referent quadrat. Because one of our primary objectives was to test the significance of "distance from the trail" for each outcome variable, the covariate QUADRAT was included in all models.

We began our data analysis with univariate regression models for all covariates and two-way interactions for each outcome variable. Data from referent quadrats (located 6.4 m from the edge of the trail) were always used as the referent values. Significant differences between quadrats on or adjacent to the trail and the referent quadrat were indicated by a significant (P ≤ 0.05) coefficient for the corresponding QUADRAT (on the trail, 0 m, 0.1 m, or 1.4 m from the trail) in the regression model. Similarly, significant relationships between outcome variables and covariates were indicated by significant coefficients for covariates.

For linear mixed-effect models, quadrats were grouped by transect number to test for significant random effects (i.e., unexplained, but correlated data between quadrats within a transect). Quadrats near feces were compared to the nearest referent quadrats. We Arcsin transformed (2* [Arcsin √Y]) all outcomes that were percentages to make them more suitable for regression analysis. We used an exponential variance function for all linear mixed-effect models in order to control for heteroscedacity. Alpha was set at 0.05 for all analyses. For Poisson regression models, we calculated *p*-values for the *t*-values calculated by S-PLUS by using an online probability calculator (J. Pezzullo; http://davidmlane.com/hyperstat/t-table.html).

## Results

Between 7 April and 5 May 2003, we sampled 120 transects (1080 quadrats) and 64 fecal piles within the Alder Canyon and Upper Willows area of Coyote Canyon. Trails ranged from 36-980 mm wide (μ = 407 mm, SD = 110) and 0-113 mm deep (μ = 48 mm, SD = 18), with a maximum trail area (width × depth) of 56 500 mm^2^. We estimated that the feral horse population in Coyote Canyon regularly used ≥ 21 km of trails within Coyote Canyon.

A total of 79 plant species were identified (14 perennial and 65 annual species; see Additional File 1), including three nonnative species: red brome grass (*Bromus madritensis spp*. *rubens*), red stem filaree (*Erodium circutarium*), and Mediterranean grass (*Schismus barbatus*). Overall, the most common species encountered was the exotic *S. barbatus*, followed by the native annual, desert pincushion (*Chaenactis fremontii*). On or along trails, the number of native species per quadrat ranged from 0-13, percent total plant cover ranged from 0-100%, and percent nonnative plant cover ranged from 0-78% (Table 1). Soil strength varied from 0-4.6 kg/cm^2 ^(4.6 kg/cm^2 ^was the maximum reading of the penetrometer; μ = 0.1.74; SD = 1.1). Near horse feces, the number of native plant species per quadrat ranged from 2-12, percent cover ranged from 10-80%, and percent nonnative cover ranged from 0-27% (Table 1).

**Table 1:** Summary of species composition and soil strength data from 120 transects centered on and adjacent to horse trails in Coyote Canyon, Anza-Borrego Desert State Park, California, USA; Spring 2003.

Location of quadrat	N	Mean percent plant cover	Mean percent nonnative plant cover	Mean number of total species	Mean number of native species	Mean number of nonnative species	Mean soil strength (kg/cm^2^)	Mean number of macro-invertebrates
Quadrat 1 (Trail)	120	13.7 (10.1)	5.7 (7.5)	4.0 (2.0)	3.0 (1.9)	1.0 (0)	2.2 (1.3)	0.63 (1.4)
Quadrat 2 (0 m)	240	31.6 (15.0)	10.4 (11.1)	5.9 (2.3)	5.0 (2.3)	1.0 (0)	1.6 (1.0)	0.52 (1.1)
Quadrat 3 (0.4 m)	240	29.0 (14.4)	10.3 (10.8)	5.7 (2.4)	4.7 (2.4)	1.0 (0)	1.7 (1.1)	0.61 (1.3)
Quadrat 4 (1.4 m)	240	30.3 (15.2)	10.9 (12.7)	5.2 (2.1)	4.3 (2.1)	1.0 (0)	NA	NA
Adjacent to feces	64	30.8 (13.7)	7.1 (6.9)	6.4 (2.2)	5.4 (2.3)	1.0 (0)	NA	NA

Quadrat 5 (Referent)	240	31.7 (15.9)	11.9 (13.5)	4.9 (2.1)	4.0 (2.1)	1.0 (0)	1.66 (1.0)	0.43 (0.8)

Standard deviations are given in parentheses. Soil strength data was not collected for quadrats at 1.4 m from the trail or adjacent to feces.

**Figure 1:**
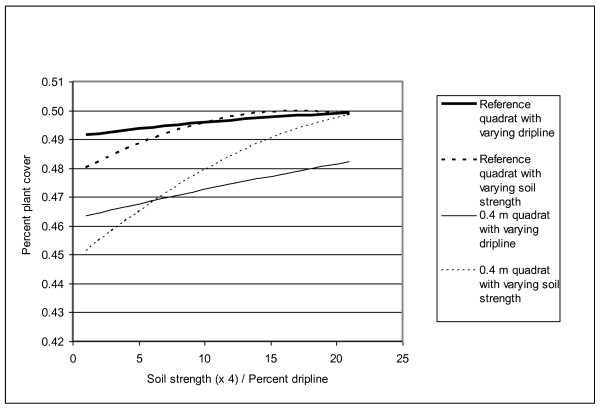
**Results from a linear mixed-effects regression model of percent vegetation cover at 0.4 m from the edge of the trail and at referent quadrats**. Graph illustrates the relationships between percent plant cover, soil strength, and dripline. After accounting for variation in dripline and soil strength, quadrats at 0.4 m from the trail had less vegetation cover than referent quadrats.

**Figure 2:**
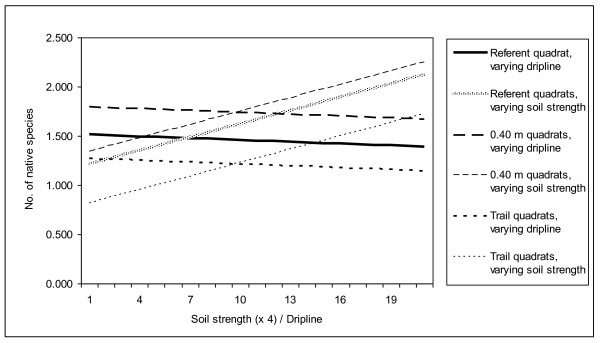
**A comparison of results from Poisson regression models of the number of native plant species at the trail center, 0.4 m from the edge of the trail, and at referent quadrats**. After accounting for differences in soil strength, plant species diversity was highest at quadrats 0.4 m from the trail.

Percent total vegetation cover was lower than referent quadrats on the trail and at 0.40 m from the trail (Table 2; Fig. 1). Our model for percent total vegetation cover accounted for significant random effects and showed that vegetation cover increased with soil strength and dripline (Table 2). Percent nonnative cover differed from the referent quadrats only on trail quadrats. Overall, our regression model showed that percent nonnative cover had a positive association with dripline and a curvilinear relationship with soil strength, with the highest percentages of nonnative cover obtained at intermediate soil strengths (Table 2). The number of native plant species per quadrat was significantly lower than referent quadrats on the trail and significantly higher than referent quadrats at 0 and 0.40 m from the trail (Table 3; Fig. 2). The number of native species per quadrat declined as dripline increased and changed curvilinearly with soil strength, again with the highest number of native species occurring at intermediate soil strengths.

**Table 2:** Linear mixed-effects regression model results from testing for differences between treatment and referent quadrats in vegetation cover, percent nonnative vegetation cover, and soil strength for horse trails at Coyote Canyon, Anza-Borrego Desert State Park, California, USA; Spring 2003.

Covariate	Coefficient (95% CI)	Back-Transformed coefficient	P-value
*Percent Total Vegetative Cover*^*a*^			
Intercept	1.14 (1.08, 1.20)	29.03 (26.21, 31.93)	<0.01
Soil strength	0.03 (0.01, 0.05)	0.022 (0.002, 0.06)	<0.01
Dripline	0.003 (0.0004, 0.005)	0.002 (3.9 × 10^-6^, 0.0006)	0.02
Quadrat^b^	---	---	<0.01^*b*^
Quadrat 1 (trail)	-0.55 (-0.61, -0.49)	-7.35 (9.08, 5.77)	<0.01
Quadrat 2 (0 m)	-0.03 (-0.09, 0.03)	-0.02 (0.22, 0.02)	0.32
Quadrat 3 (0.4 m)	-0.09 (-0.15, -0.03)	-0.19 (0.53, 0.02)	<0.01
Quadrat 4 (1.4 m)	-0.09 (-0.27, 0.10)	-0.19 (1.85, 0.24)	0.35
Quadrat 5 (Referent)	---	--	---
Random effects	0.12 (0.09, 0.15)	0.12 (0.21, 0.59)	<0.01
			
*Percent nonnative cover*^*a*^			
Intercept	0.38 (0.30, 0.47)	3.60 (2.23, 5.31)	<0.01
Soil strength	0.15 (0.08, 0.21)	0.53 (0.16, 1.13)	<0.01
Soil strength^2^	-0.03 (-0.04, 0.21)	-0.02 (0.04, 1.13)	0.01
Dripline	0.003 (0.0002, 0.005)	0.0002 (1.0 × 10^-6^, 0.0006)	0.03
Quadrat^b^	---	---	<0.01^b^
Quadrat 1 (trail)	-0.17 (-0.23, -0.11)	-0.72 (1.32, 0.30)	<0.01
Quadrat 2 (0 m)	-0.02 (-0.07, 0.04)	-0.01 (0.14, 0.03)	0.51
Quadrat 3 (0.4 m)	-0.04 (-0.09, 0.02)	-0.03 (0.20, 0.01)	0.20
Quadrat 4 (1.4 m)	-0.09 (-0.23, 0.06)	-0.19 (1.35, 0.09)	0.25
Quadrat 5 (Referent)	---	--	---
Random effects	0.18 (0.15, 0.22)	0.79 (0.53, 1.16)	<0.01
			
*Soil strength*			
Intercept	2.44 (2.148, 2.727)	88.19 (77.37, 95.82)	<0.01
Rain	-0.73 (-0.95, -0.51)	12.74 (6.36, 20.92)	<0.01
Trail Area	-2.1 × 10^-5 ^(-3.2 × 10^-5^, -0.0010)	-1.0 × 10^-5 ^(3.0 × 10^-10^, 3.0 × 10^-9^)	<0.01
Dripline	-0.0045 (-0.0081, -0.0010)	-0.0005 (3.0 × 10^-5^, 0.002)	0.01
Horse sign (present)	-0.15 (-0.29, -0.01)	-0.56 (0.002, 0.02)	0.04
Horse sign (Referent)	---	---	---
Quadrat^b^	---	---	<0.01^*b*^
Quadrat 1 (trail)	0.48 (0.27, 0.68)	0.24 (1.81, 11.12)	<0.01
Quadrat 2 (0 m)	-0.15 (-0.28, -0.01)	-0.56 (0.002, 1.95)	0.03
Quadrat 3 (0.4 m)	-0.15 (-0.28, -0.02)	-0.56 (0.01, 1.95)	0.02
Quadrat 4 (1.4 m)	NA	NA	NA
Quadrat 5 (Referent)	---	---	---
Random effects	0.48 (0.45, 0.64)	0.48 (0.45, 0.64)	<0.01

^a^ Arcsin transformed; ^b^ Likelihood ratio test to determine the significance of the overall covariate.

**Table 3:** Results from Poisson regression models used to test for differences between treatment and referent quadrats in variables related to feral horse impacts at Coyote Canyon, Anza-Borrego Desert State Park, California, USA; Spring 2003.

Covariate	Coefficient (95% CI)	Back-Transformed Coefficient (95% CI)	P-value
*Number of native species*			
Intercept	1.20 (1.07, 1.33)	3.29 (2.91, 3.78)	<0.01
Soil strength	0.22 (0.10, 0.33)	1.24 (1.11, 1.39)	<0.01
Soil strength^2^	-0.03 (-0.06, -0.01)	-0.97 (0.94, 0.99)	<0.01
Dripline	-0.01 (-0.01, -0.00)	-0.99 (0.99, 1.0)	<0.01
Quadrat^b^	---	---	<0.01
Quadrat 1 (trail)	-0.36 (-0.49, -0.23)	-0.96 (0.61, 0.80)	<0.01
Quadrat 2 (0 m)	0.21 (0.12, 0.30)	1.23 (1.12, 1.35)	<0.01
Quadrat 3 (0.10 m)	0.16 (0.07, 0.26)	1.18 (1.07, 1.29)	<0.01
Quadrat 4 (1.4 m)	-0.01 (-0.30, 0.27)	-0.99 (0.74, 1.31)	0.93
Quadrat 5 (Referent)	---	---	---
			
*Number of macroinvertebrates*			
Intercept	-0.71 (-1.04, -0.38)	-0.49 (0.35, 0.68)	<0.01
Soil strength	-0.27 (-0.37, -0.17)	-0.76 (0.69, 0.98)	<0.01
Day	0.03 (0.01, 0.06)	1.03 (1.01, 1.06)	<0.01
Quadrat^a^	---	---	0.10^*c*^
Quadrat 1 (trail)	0.54 (0.24, 0.85)	1.72 (1.27, 2.33)	<0.01
Quadrat 2 (0.00 m)	0.24 (-0.03, 0.51)	1.27 (0.97, 1.67)	0.08
Quadrat 3 (0.40 m)	0.38 (0.12, 0.64)	1.46 (1.13, 1.90)	<0.01
Quadrat 4 (1.4 m)	NA	NA	NA
Quadrat 5 (Referent)	---	---	---

Regression models were also used to quantify associations between covariate and outcome variables.

^a^Likelihood ratio test to determine the significance of the overall covariate.

Trails had higher soil strength than referent quadrats, while quadrats at 0.0 and 0.4 m had lower soil strength than referent quadrats (Table 2). Our model for soil strength accounted for random effects among transects and showed that soil strength decreased with rain, trail area, dripline, and horse sign (i.e., hoof prints or horse feces). In quadrats near horse feces, we found higher native plant diversity (number of native plant species) than at nearby referent quadrats (Table 4). Neither total percent vegetation cover nor percent nonnative vegetation cover differed near horse feces. We found higher numbers of macroinvertebrates on the trail and at 0.40 m from the trail than at referent quadrats (Table 3). The number of macroinvertebrates found per quadrat decreased with increasing soil strength and varied significantly among days.

**Table 4:** Results from linear mixed-effects or Poisson regression models used to test for and quantify differences in total vegetation cover, percent nonnative cover, and number of native species between quadrats near horse feces and referent quadrats at horse trails in Coyote Canyon, Anza-Borrego Desert State Park, California, USA; Spring 2003.

Outcome variable	Covariate	Coefficient (CI)	P-value
*Percent total vegetative cover*			
	Intercept	1.07 (0.99, 1.16)	<0.01
	Referent quadrat	--	--
	Quadrat near feces	0.07 (0.0, 0.17)	0.23
			
*Percent nonnative cover*			
	Intercept	0.53 (0.44, 0.62)	<0.01
	Referent quadrat	--	--
	Quadrat near feces	-0.07 (-0.16, 0.02)	0.13
			
*Number of native species*			
	Intercept	1.49 (1.38,1.61)	<0.01
	Referent quadrat	--	--
	Quadrat near feces	0.20 (0.04, 0.35)	<0.01

Trail quadrats differed significantly from referent quadrats for all five parameters examined (Table 5). Immediately adjacent to the trail, the only significant differences from referent quadrats were the number of native plant species and soil strength. Quadrats located 0.4 m from the edge of the trail differed relative to referent quadrats for four parameters (i.e., decreased total vegetation cover, higher species diversity, more macroinvertebrates, and reduced soil strength). There was no evidence of differences in vegetation characteristics at 1.4 m from the trail, compared to referent quadrats. Assuming an average trail width of approximately 40 cm, the total area impacted along horse trails is estimated to be approximately 1.2 m in width.

**Table 5:** Summary of significant differences in parameters between sampling quadrats and referent quadrats.

	Trail Quadrat	0.0 m Quadrat	0.4 m Quadrat	1.4 m Quadrat	Quadrat near feces
Percent plant cover	↓	-	↓	-	-
Percent nonnative cover	↓	-	-	-	-
No. of native plant species	↓	↑	↑	-	↑
No. of macroinvertebreates	↑	-	↑	NA	NA
Soil strength	↑	↓	↓	NA	NA

Arrows indicate significant increases or decreases relative to referent quadrats and dashes indicate no difference from referent quadrats. Referent quadrats were located at 6.4 m from the edge of trails used by feral horses in Coyote Canyon, Anza-Borrego Desert State Park, California, USA; Spring 2003.

## Discussion

One of the most interesting results from this study is an estimate of the area subjected to indirect biotic impacts associated with feral horse trail use. If all trails were similarly impacted, at least 25 km^2 ^(1.2 m × 21 000 m) of habitat within greater Coyote Canyon was affected by feral horse trail use. In areas inhabited by large feral horse populations across the western U.S., the geomorphologic impact of feral horses through erosion and reduced plant cover may be substantial.

The impacts of feral horses on trails were much different than impacts to areas adjacent to trails. Trails were characterized as having significantly compacted soil, low plant cover, a high percentage of bare ground, and low species diversity (Table 5). In contrast, areas adjacent to trails (0-0.4 m) had increased plant diversity and lower soil strength. Overall, our findings on and along trails used by feral horses are consistent with the anticipated outcome of large mammal trampling in an arid environment: reduced vegetation cover, compacted soils, and in cases of intermediate intensity disturbance, increased plant species diversity [3,22].

High plant diversity near trails used by feral horses is consistent with the intermediate disturbance hypothesis [23] and the model presented by Milchunas et al. [22]. Increases in plant species diversity with low-density livestock use is a well-documented phenomenon, although the effect is predicted to be smallest in arid and semiarid environments [9,22,24]. Our results corroborate the pattern of increased plant diversity at small spatial scales with light to moderate levels of disturbance in an arid environment.

The deposition of horse feces (which contain seeds, moisture, and nutrients) is another potential mechanism that may enhance plant diversity and cover in habitat used by feral horses. The role of herbivores in dispersing seeds is well established [25], and several studies have shown that horses pass large numbers of seeds through their digestive tract [26,27]. We found that quadrats near feces had significantly higher plant diversity than all other locations (Table 5). Although we hypothesized that feral horse feces may improve conditions for nonnative species, we found the least amount of nonnative plant cover near feces (Table 1).

Our results suggest that feral horse trampling in an arid environment contributes to both soil compaction and erosion. Hendee and Dawson [28] found that controlled amounts of use by horses on trails caused increased soil compaction. In contrast, DeLuca et al. [29] reported that horse use loosened soil and made it more susceptible to erosion. In our study, soil often appeared to have been chiseled (by hooves), washed, and/or blown from the trail and collected along the edges of the trail, which could explain the significantly looser soil we found at quadrats 0 and 0.4 m from the trail relative to referent quadrats (Table 5).

While our study found compacted soils (relative to reference quadrats) only on trails and not adjacent to trails, an important caveat to our findings is that the referent quadrats in our study experienced some level of horse use and historic cattle grazing. Comparing our study area to a pristine, non-horse area may show that soils in nearly all areas used by feral horses were compacted. Beever [30] and Beever and Herrick [7] found that horse-grazed sites had greater soil strength than horse-excluded areas. Additionally, it is possible that grazing impacts were found throughout our study area and therefore minimized the differences between trails and referent quadrats for all five parameters we measured. In general, herbivory on a large scale ultimately selects for more grazing-tolerant species [31]; therefore, grazing-sensitive plant species in Coyote Canyon may have been eliminated from the local plant assemblage during a past period of cattle grazing. This idea of long-term and widespread impacts is consistent with the nonnative grass *S. barbatus*, which is often associated with disturbed areas [19], being the most abundant plant in our study area.

Our finding of greater macroinvertebrate abundance on the trail and at 0.4 m from the trail (where vegetation cover was also reduced) could be interpreted at least two ways. First, greater macroinvertebrate abundance may reflect superior habitat conditions for the species we observed. An alternative interpretation of our macroinvertebrate data is that visibility was higher in areas having less vegetation, hence the correlation between reduced vegetation cover and greater macroinvertebrate abundance. We recommend that future studies that incorporate macroinvertebrate monitoring focus on specific taxa, with known habitat requirements. For example, Beever and Herrick [7] reported that ant mounds were more abundant at sites where feral horses had been removed.

## Conclusion

Our results show that feral horses may cause substantial indirect geomorphic changes. This finding is consistent with the idea of using feral horses as "ecosystem engineers" in highly-managed conservation areas [15]. Understanding and quantifying the impacts of feral horses on their environment will help wildlife managers to maintain the biotic integrity of habitat used by feral horses, as required by the Wild Free-Roaming Horse and Burro Act of 1971. If feral horse populations are maintained within habitat of threatened or endangered species known to be sensitive to loss of vegetative cover (e.g., lizards, grassland birds, and some small mammals), precautions should be taken to avoid impacts to these species. The results from our study should be extrapolated carefully because the impacts of grazing on arid environments are highly influenced by animal density and abiotic factors [22,32]. Also, this study was conducted in upland areas, and the impacts of horses in riparian areas may have much more significant impacts [33].

## Authors' contributions

SO designed the study, drafted the manuscript, and conducted the fieldwork with LH. EA and ER assisted with study design, analyses and manuscript review. WB conceived the idea, supervised the research and revised the manuscript. All authors have approved the final manuscript.

## Supplementary Material

Additional file 1 Plant species identified in and adjacent to transects during Spring 2003 in Upper Willows and Alder Canyon within Coyote Canyon, Anza-Borrego Desert State Park, California, USA. Click here for file
