# Supplementary material for: Impacts of feral horses on a desert environment
Source: BMC Ecol. 2009 Nov 10;9:22. doi: 10.1186/1472-6785-9-22 (PMC2781800; doi:10.1186/1472-6785-9-22)
Supplement: Additional file 1 — Plant species identified in and adjacent to transects during Spring 2003 in Upper Willows and Alder Canyon within Coyote Canyon, Anza-Borrego Desert State Park, California, USA. [file 1472-6785-9-22-S1.doc]

**Additional File 1** - Plant species identified in and adjacent to transects during Spring 2003 in Upper Willows and Alder Canyon within Coyote Canyon, Anza-Borrego Desert State Park, California, USA.

| Scientific Name | Common Name | Native Annual | Native Perennial | Non-native |
| --- | --- | --- | --- | --- |
| *Abronia villosa* var. *villosa* | Sand Verbena | x |  |  |
| *Acacia greggii* | Catclaw Acacia |  | x |  |
| *Ambrosia dumosa* | White Bur-sage/Burrobush |  | x |  |
| *Ambrosia salsola* var. *salsola* | Cheesebush |  | x |  |
| *Amsinckia menziesii* var. *menziesii* | Rancher's Fireweed | x |  |  |
| *Bromus rubens* | Foxtail Chess |  |  | x |
| *Calyptridium monandrum* | Common Calyptridium | x |  |  |
| *Camissonia californica* | False Mustard | x |  |  |
| *Camissonia pallida* ssp. *pallida* | Pale Yellow Sun Cup | x |  |  |
| *Centrostegia thurberi* | Thurber's Spineflower | x |  |  |
| *Chaenactis fremontii* | Desert Pincushion | x |  |  |
| *Chorizanthe brevicornu* var. *brevicornu* | Brittle Spineflower | x |  |  |
| *Chorizanthe xanti* var. *leucotheca** | White-bracted Spineflower | x |  |  |
| *Chorizanthe watsonii* | Watson's Spineflower | x |  |  |
| *Crassula connata* | Pygmyweed | x |  |  |
| *Cryptantha circumscissa* | Western Cryptantha | x |  |  |
| *Cryptantha micrantha* | Purple-root Cryptantha | x |  |  |
| *Cryptantha nevadensis* | Nevada Cryptantha | x |  |  |
| *Cryptantha pterocarya* var. *cycloptera* | Single-wing Cryptantha | x |  |  |
| *Cryptantha recurvata* | Recurved Cryptantha | x |  |  |
| *Cylindropuntia ganderi* var. *ganderi* | Gander's Cholla |  | x |  |
| *Dichelostemma capitatum* ssp. *capitatum* | Blue Dicks |  | x |  |
| *Emmenanthe penduliflora* var. *penduliflora* | Whispering Bells | x |  |  |
| *Ephedra californica* | California Ephedra |  | x |  |
| *Erodium cicutarium* | Red-stem Filaree |  |  | x |
| *Eriastrum diffusum* | Miniature Woolly Star | x |  |  |
| *Eriastrum eremicum* ssp. *eremicum* | Desert Woolly Star | x |  |  |
| *Eriogonum fasciculatum* var. *polifolium* | Mountain Buckwheat |  | x |  |
| *Ericameria paniculata* | Punctate/Black-stem Rabbitbrush |  | x |  |
| *Eriogonum gracile* var. *incultum* | Slender Buckwheat | x |  |  |
| *Eriogonum thurberi* | Thurber's Buckwheat | x |  |  |
| *Eriophyllum wallacei* | Wallace's Woolly Daisy | x |  |  |
| *Eschscholzia minuiflora* | Pygmy Gold-poppy | x |  |  |
| *Eucrypta chrysanthemifolia* var. *bipinnatifida* | Spotted Hideseed | x |  |  |
| *Gilia stellata* | Star Gila | x |  |  |
| *Hilaria rigida* | Big Galleta |  | x |  |
| *Lastarriaea coriacea* | Lastarriaea | x |  |  |
| *Layia glandulosa* | White Layia | x |  |  |
| *Leptosiphon lemmonii* | Lemmon's Linanthus | x |  |  |
| *Linanthus* sp. |  | x |  |  |
| *Loeseliastrum matthewsii* | Desert Calico | x |  |  |
| *Loeflingia squarrosa* var. *squarrosa* | California Loeflingia | x |  |  |
| *Logfia depressa* | Dwarf Filago | x |  |  |
| *Lotus* sp. |  | x |  |  |
| *Lotus scoparius* var. *brevialatus* | Deerweed |  | x |  |
| *Lotus strigosus* | Bishop's/Strigose Lotus | x |  |  |
| *Lupinus bicolor* | Miniature Lupine | x |  |  |
| *Lupinus concinnus* | Bajada Lupine | x |  |  |
| *Lupinus sparsiflorus* | Coulter's Lupine | x |  |  |
| *Malacothrix glabrata* | Desert Dandelion | x |  |  |
| *Mentzelia albicaulis* | Kuha | x |  |  |
| *Muilla maritima* | Common Muilla |  | x |  |
| *Nama demissum* var. *demissum* | Purple Mat | x |  |  |
| *Nemocladus* sp. |  | x |  |  |
| *Pectocarya penicillata* | Winged Pectocarya | x |  |  |
| *Pectocarya recurvata* | Recurved Pectocarya | x |  |  |
| *Phacelia distans* | Wild Heliotrope | x |  |  |
| *Pholistoma membranaceum* | White Fiesta Flower | x |  |  |
| *Plagiobothrys arizonicus* | Arizona Popcornflower | x |  |  |
| *Plantago patagonica* | Desert Plantain | x |  |  |
| *Prosopis glandulosa* var. *torreyana* | Honey Mesquite |  | x |  |
| *Salvia columbariae* | Chia | x |  |  |
| *Schismus barbatus* | Mediterranean Schismus |  |  | x |
| *Stephanomeria exigua* ssp. *exigua* | Small Wreath Plant | x |  |  |
| *Stylocine gnaphaloides* | Everlasting Nest Straw | x |  |  |
| *Thysanocarpus curvipes* | Lacepod/Fringepod | x |  |  |
| *Thamnosma montana* | Turpentine Broom |  | x |  |
| *Vulpia octoflora* var. *hirtella* | Tufted Fescue | x |  |  |

* rare, threatened, or endangered in California and elsewhere, California Native Plants Society Inventory of Rare and Endangered Plants (7th edition online): <http://cnps.web.aplus.net/cgi-bin/inv/inventory.cgi>

Plants were identified using Jepson Manual: higher plants of California. James C. Hickman, editor, 1993, and  Flora of North America, published volumes, (FNA online): <http://hua.huh.harvard.edu/FNA>  [for updated taxonomy].
